# 789 Six Year Experience with Autologous Skin Cell Suspension for Burn Wounds

**DOI:** 10.1093/jbcr/irae036.330

**Published:** 2024-04-17

**Authors:** William West, Sarah Moffitt, Timothy Nehila, Rithvic Jupudi, Kristina Buller, Nicole K Le, Kristen Whalen, Jared Troy, Jake Laun

**Affiliations:** Morsani College of Medicine USF, Tampa, FL; Morsani College of Medicine USF, Tampa, FL; Morsani College of Medicine USF, Tampa, FL; Morsani College of Medicine USF, Tampa, FL; Morsani College of Medicine USF, Tampa, FL; Morsani College of Medicine USF, Tampa, FL; Morsani College of Medicine USF, Tampa, FL; Morsani College of Medicine USF, Tampa, FL; Morsani College of Medicine USF, Tampa, FL

## Abstract

**Introduction:**

Autologous skin cell suspensions (ASCS) minimize the donor site required for addressing partial and full thickness burns. ASCS is currently FDA approved for use in combination with meshed split thickness skin grafts (STSGs) for full-thickness thermal burns in pediatric and adult patients. Besides the initial clinical trials of ASCS and STSG use for burn wounds, there are minimal studies reporting outcomes of their use. Here, we present our experience using ASCS in the past six years. We hypothesized that ASCS and STSG would result in a low reoperation and infection rate.

**Methods:**

Retrospective review of patients seen at an American Burn Association verified burn center between 2017 and 2023 identified 15 patients treated with ASCS overlying STSG. Data collected included patient demographics, burn characteristics, surface area of ASCS usage, and patient outcomes. The primary outcome of interest was the requirement for reoperation following ASCS use. Secondary outcomes included hospital length of stay (LOS), ICU LOS, infection, and the necessity for scar revision via surgery or laser treatment. Data was analyzed using descriptive statistics with categorical variables presented as frequencies and percentages and continuous variables reported as medians and ranges.

**Results:**

The median age of patients treated with ASCS was 36 years (13-67 years). The median BMI was 27.4 (17.7-35.4) and median total body surface area (TBSA) affected by burn was 18% (5.75-69.5%). Six patients (40%) had full thickness in addition to partial thickness burns. Most burns, 11 (73%) were the result of flames, although there were three grease burns and one friction burn. The median time to ASCS application was 10 days (1-39 days) and the median number of ASCS application sites was 2 (1-6). Most ASCS sites were on the upper or lower extremities or the torso; however, two patients had ASCS applied to their feet while another patient had it applied to their genitalia. Median ASCS surface area was 2,464 cm2 (289-20,000 cm2). Median LOS was 23 days (8-125 days) and median ICU LOS was 3 days (0-94 days). Four patients (26.7%) required reoperation on sites of ASCS application with the median time to reoperation being 40.5 days (28-66 days). Three patients (20%) required scar contracture surgery and three (20%) received laser treatments. There were no cutaneous infections in our cohort.

**Conclusions:**

ASCS was effectively used to treat both partial thickness and full thickness burns and burns due to flame, grease, and friction. There did not seem to be any relationship between the necessity for reoperation after ASCS and the burn depth, etiology, surface area, or location.

**Applicability of Research to Practice:**

Clinicians can look to our study for potential outcomes when using ASCS with STSG in a variety of situations.

